# The association of area-level social class and tobacco use with adverse breast cancer characteristics among white and black women: evidence from Maryland, 1992–2003

**DOI:** 10.1186/s12942-015-0007-7

**Published:** 2015-04-01

**Authors:** Ann C Klassen, Aaron Pankiewicz, Stephanie Hsieh, Abigail Ward, Frank C Curriero

**Affiliations:** Department of Community Health and Prevention, Drexel University School of Public Health, 3215 Market Street, 4th Floor, Philadelphia, PA 19104 USA; Department of Health, Behavior and Society, Johns Hopkins Bloomberg School of Public Health, Baltimore, MD USA; Department of Epidemiology, Johns Hopkins Bloomberg School of Public Health, Baltimore, MD USA; Department of Biostatistics, Johns Hopkins Bloomberg School of Public Health, Baltimore, MD USA

**Keywords:** Race, Social Class, Tobacco, Breast Cancer, Disparities

## Abstract

**Background:**

In breast cancer, worse disease characteristics are associated with fewer social resources and black race. However, it is unknown whether social gradients have similar impact across race, and whether behaviors, including tobacco use, may explain a portion of the social gradient.

**Methods:**

We modeled relationships between area-level social class, tobacco spending and tumor characteristics, using 50,062 white and black cases diagnosed from 1992–2003 in Maryland, a racially and economically diverse state on the east coast of the United States. Multi-level models estimated the effect of area-level social class and tobacco consumption on tumor grade, size, and stage at diagnosis.

**Results:**

Adjusting for race, age and year of diagnosis, higher social class was associated with lower risk for tumors with histological grade 3 or 4 (O.R. 0.96, 95% C.I. 0.94,0.99), those diagnosed at SEER stage 2 or later (O.R. 0.89, 95% C.I. 0.86, 0.91), and tumor size >2 cm (O.R. 0.87, 95% C.I. 0.84, 0.90). Higher tobacco spending was associated with higher risk for higher grade (O.R. 1.01, 1.00, 1.03) and larger tumors (O.R. 1.03, 95% C.I. 1.01, 1.06), but was not statistically significantly related to later stage (O.R. 1.00, 95% C.I. 0.98, 1.02). Social class was less protective for black women, but tobacco effects were not race-specific.

**Conclusions:**

Results suggest that in one U.S. geographic area, there is a differential protection from social class for black and white women, supporting use of intersectionality theory in breast cancer disparities investigations. Area-level tobacco consumption may capture cases’ direct use and second hand smoke exposure, but also may identify neighborhoods with excess cancer-related behavioral or environmental exposures, beyond those measured by social class. Given the growing global burden of both tobacco addiction and aggressive breast cancer, similar investigations across diverse geographic areas are warranted.

## Background

Breast cancer is the most commonly diagnosed cancer in U.S. women [1], and is now the leading cause of cancer death for women globally as well [2]. Historically, breast cancer has been considered a “disease of affluence”, and incidence rates remain highest in high income countries, and among women of higher social class [3], due in part to a range of lifestyle factors, including nutrition and development, physical activity, childbearing and lactation practices [3-6]. However, disease burden differs significantly by geography, ethnicity and socio-economic status. For example, the proportion of breast cancers which are younger-onset and more aggressive is higher in Africa [7] and other non-industrialized regions. In the United States, African-American race [8-12] and low SES [13,14] are consistently associated with more adverse disease characteristics, including advanced stage at diagnosis, larger tumor size [10], and more aggressive tumor biology [9,13].

Although ethnic disparities are often speculated to be driven at least in part by social and economic resource differentials [15], there is incomplete understanding of the race-specific role of social resources on breast cancer outcomes, and whether specific behavioral correlates of social class actually drive biological outcomes. In a comprehensive review of 90 studies which examined the relationship between social class and breast cancer, the majority (n = 55) focused on breast cancer incidence, twenty-three examined breast-cancer related mortality, and only twelve explored the relationship between social class and breast cancer biology or disease characteristics, with four of those from Europe, three from Asia, and five from the United States.

Breast cancer disparities, at their most essential, represent the biological manifestation of a myriad of physical, environmental, social and behavioral differences between women. Research using surveillance data to consider both individual and community influences on breast cancer patterns can provide a complementary approach to investigations within specific clinical populations. The strength of surveillance data are their complete enumeration of all cases within a geographic area over time; however, as they represent a secondary use of data abstracted from clinical records, they typically lack the type of detailed social and demographic information desirable for social and behavioral science investigations.

However, the growing sophistication of geographically referenced analysis allows linkage of population-based surveillance data, by geography, to attributes of the neighborhood environment [16-18]. Geographic analyses of disease patterns may reveal synergistic effects of multiple social and behavioral influences which may not come to light in studies of single variables outside the geographic context. Recent growth in the availability of geographically referenced data, including Census and consumer expenditure-based “geodemographics” [19,20], allow health researchers to describe and understand more fully the nature of communities with excess health burden. Such data can serve as surrogates when individual-level behavioral data are not available, and also identify contextual characteristics (group behaviors and norms, crime, green space, food deserts, etc.) which influence all members of communities [21-23].

Geographically referenced analyses are limited to ecological associations, and cannot establish a causal link between individual behavior and risk. As well, they can only establish cross-sectional associations at time of diagnosis, without providing insight into social environments during earlier, likely critical periods for cancer initiation across the life course. However, exploratory studies in large geographically referenced datasets may identify the most promising relationships between social and behavioral factors and adverse cancer outcomes. This can help prioritize specific hypotheses to test in more resource-intensive efforts such as primary data collection and longitudinal cohort studies.

The purpose of this analysis is to explore the relationship between social class, tobacco use and adverse breast cancer characteristics among white and African-American women diagnosed with breast cancer in Maryland between 1992 and 2003. Both active smoking and exposure to environmental tobacco smoke (ETS) have been studied extensively in regard to breast cancer incidence, with mixed results [24-27]. However, less is known about the relationship between tobacco exposure and breast cancer disease characteristics. Our comprehensive literature review found no studies exploring race- and social class-specific patterns of tobacco exposure and breast cancer. There is an increasing concentration of tobacco use among communities of lower socioeconomic status [28], and globally, both a growing burden of tobacco addiction, and increased incidence of breast cancer. Given the possible synergistic effects of tobacco exposure with other socially pattern risk related to diet, physical activity, and environmental exposures, examining social class and tobacco use in geographic context may provide additional insight into the causes of excess breast cancer burden [29-31].

## Results

During the years 1992 to 2003, a total of 54,842 cases of breast cancer were reported to the Maryland Cancer Registry. Of these, 1507 records were not able to be released to the researchers (939 from vital statistics/death records only, 552 reported back to Maryland from states not allowing research use of shared data, and 16 from the Veterans Administration). An additional 2573 records were not used for this analysis because they were male breast cancers (n = 482), non-white, non-black cases (637 Asian, 52 Native American), or missing race (n = 1402). Of the 50762 records available for the analysis, 50719 were determined to have adequate covariate data for analysis, and were retained.

Using residential address as listed in the Maryland Cancer Registry record, 43,081 cases were geocoded to a point location, representing an 85% geocoding success rate. An additional 7233 cases were determined to have legitimate Maryland addresses, and were assigned to a point location within their zip code, based on our previously published [32] imputation algorithm and Census data on age-, race- and gender-specific population distributions within their zip code. Thus, a total of 50314 cases were determined to represent Maryland residents by address, and were assigned to a location within the State of Maryland. Cases resided in 3624 of 3678 Maryland block groups (98%).

After construction and linkage of the block group-level Census and CES data, 33 of the block groups were found to have missing or extreme Census or CES values (i.e., unfeasibly high per capita tobacco spending due to Census estimates of only a few residents in a block group). After these were dropped, the final dataset used for multivariate analyses contained 50,062 cases residing in 3591 Maryland block groups. These data represent 99.5% of study–eligible cases with complete records who were determined to be Maryland residents, and utilize 97.6% of Maryland 2000 Census block groups.

Table 1 describes the distribution of individual and block group level characteristics for the group as a whole, and also by racial group. The majority of the cases overall and within both white and black racial groups were over age 55, although more of the African American cases were younger. There was an increase in the total number of cases diagnosed annually across the twelve year time period, and an increase in the proportion of cases who were African American. Among the 69% of cases for whom histological grade was reported, more cases overall, and more white cases received the less aggressive grades of 1 or 2; however, more African American cases received grades of 3 or 4, representing a potentially more aggressive cancer. Of the 93% of cases with a SEER Summary stage at diagnosis [33] recorded, the majority had their disease diagnosed at the in-situ or local stage, although a greater proportion of African American cases had more advanced stage at time of diagnosis.

**Table 1:** **Distribution of individual and area-level characteristics by race, among white and black women with breast cancer, reported to the Maryland cancer registry, 1992-2003**

			**Race/Ethnicity of cases**	
	**Blockgroups (n = 3,591)**	**Cases (n = 50,062)**	**Whites (n = 39,116)**	**Blacks (n = 10,946)**
**Individual level**	%	%	%	%
Age at Dx				
21-35		2.8	2.3	4.7
36-55		36.9	34.5	45.7
56-75		43.3	44.6	38.5
76-106		17.0	18.6	11.1
Year of Dx				
1992-1995		29.7	30.3	27.5
1996-1999		34.7	34.8	34.4
2000-2003		35.6	34.9	38.1
Grade				
1,2		39.2	41.4	31.2
3,4		29.3	27.1	37.3
No grade		31.5	31.5	31.5
Stage at Dx				
In-situ, local		66.4	68.3	59.9
Regional, Distant		26.8	25.2	32.2
No stage		6.8	6.5	7.9
Tumor Size (1999–2003 only)		(n = 21,573)	(n = 16,570)	(n = 5,003)
<20 mm		47.9	50.6	38.8
20+ mm		32.1	30.2	38.2
Not reported		20.0	19.2	23.0
**Area-Level**				
High School Graduation Rate				
26-69%	20.4	14.2	10.1	28.6
70-79%	21.0	19.4	18.8	21.8
80-89%	31.6	33.3	33.8	31.5
90-100%	27.0	33.1	37.3	18.1
Employment				
52-84%	2.1	1.1	0.2	4.0
85-89%	3.4	1.9	0.6	6.9
90-94%	12.8	9.9	6.4	22.4
95-100%	81.7	87.1	92.8	66.7
White Collar Employment				
13-49%	18.7	13.2	10.9	21.7
50-64%	33.5	30.0	28.4	35.9
65-74%	22.4	23.8	23.5	24.8
75-98%	25.4	33.0	37.3	17.6
Per Capita Income (in $1000)				
4-14	18.9	12.7	7.4	31.7
15-19	23.9	21.0	19.9	25.0
20-29	39.7	42.1	44.2	34.6
30-107	17.6	24.1	28.4	8.7
Tobacco				
$123-599	29.5	33.0	27.7	51.8
$600-699	19.6	18.9	16.7	27.0
$700-799	17.0	16.7	18.0	11.7
$800-2442	33.9	31.4	37.6	9.4

Differences in distribution between white and black cases, all significant at p < 0.001, chi square test.

The final individual level disease characteristic, tumor size, was only consistently recorded during the years 1999–2003. Of the 80% of cases during these years with reported tumor size, the majority of tumors were two centimeters or less, although the race-specific analyses show this is not the case for black women. In summary, African American cases were more likely in bivariate analyses to have more aggressive, larger tumors diagnosed at a later stage.

Table 1 also shows that a slightly higher proportion of black cases than white cases have no tumor size reported (23% versus 19%) and are missing stage information (7.9 versus 6.5%), but there are no differences in missing histological grade (31.5% for both racial groups). We also explored age-specific patterns (data not shown) and found that women age 60 and older were slightly less likely than those 59 or younger to have no tumor size reported (19.4% versus 20.7%), but more likely to have unreported tumor grade (33% versus 29%), and to be unstaged (7.6% versus 6%).

When examining the distribution of social class variables at the Census block group level in Maryland, the relatively affluence of the State is apparent, and we also see that breast cancer cases are somewhat more concentrated in the block groups with higher social resources. For example, only 20% of the individual block groups used in the analysis are communities where less than 70% of adult residents achieved a high school education, and only 14% of the cases live within those block groups. Similarly, 82% of block groups and 87% of cases have block group-level employment rates of 95% or greater. High rates of white collar employment are not as common, likely because the State of Maryland also contains significant areas of relatively wealthy agricultural and industrial employment; however, only 10.9% of white cases, and 21.7% of black cases, reside in block groups where the majority of workers are not in white collar occupations. A greater proportion of black cases reside in block groups with lower average per capita income, with 56.7% of black cases, but only 27.3% of white cases, living in block groups with an average per capita income of less than $20,000. Estimates of annual household tobacco spending range from $123 to $2442, and a greater proportion of black cases live in block groups with lower estimated household tobacco spending. All differences in distributions between white and black cases were statistically significant at p < 0.001, based on a Chi Square test.

Older women and women diagnosed later in the twelve year period were less likely to have grade 3 or 4 tumors, while African-American women were significantly more likely to have an aggressive tumor (Table 2, Model 1). In addition to these individual factors, higher area-level social class was protective of aggressive tumor histology (Model 2). In Model 3, a significant interaction term for social class and black race indicates the protective effect of social class is reduced for black women. Model 4 indicates that higher area-level tobacco spending contributes additional independent risk for aggressive grade, although the effect is relatively modest, with an increase of 1% in the odds of aggressive tumor histology associated with a $100 increase in average annual household spending on tobacco, and only marginally significant (p = 0.05). There is no statistically significant interaction between tobacco spending and race (Model 5), suggesting that this effect does not differ for white and black cases.

**Table 2:** **Multi-level random effects logistic regression model of individual and area-level factors associated with aggressive histological grade at diagnosis for breast cancer among white and black women in Maryland, 1992–2003 (34,310 women in 3568 census blockgroups, Maryland cancer registry)**

	**Model 1**		**Model 2**		**Model 3**		**Model 4**		**Model 5**	
Fixed Effects	O.R.	95% CI	O.R.	95% CI	O.R.	95% CI	O.R.	95% CI	O.R.	95% CI
Intercept	0.71	(0.70,0.74)	0.72	(0.70,0.74)	0.72	(0.70,0.74)	0.64	(0.56,0.73)	0.63	(0.53,0.71)
Individual Level										
Age	0.98	(0.98,0.98)	0.98	(0.98,0.98)	0.98	(0.98,0.98)	0.98	(0.98,0.98)	0.98	(0.98,0.98)
Black Race	1.69	(1.60,1.78)	1.65	(1.56,1.74)	1.69	(1.59,1.79)	1.73	(1.62,1.85)	1.71	(1.59,1.83)
Year of Diagnosis	0.95	(0.95,0.96)	0.96	(0.95,0.96)	0.96	(0.95,0.96)	0.96	(0.95,0.96)	0.96	(0.95,0.96)
Census Blockgroup Level										
Social Class			0.96	(0.94,0.99)	0.94	(0.92,0.97)	0.96	(0.93,0.99)	0.97	(0.93,1.00)
Class*Black Race					1.08	(1.03,1.15)	1.06	(1.01.1.13)	1.06	(0.99,1.12)
Tobacco Spending (per $100)							1.01	(1.00,1.03)	1.02	(1.00,1.04)
Tobacco*Black Race									0.98	(0.94,1.02)
Random Effects	*Estimate*	*P Value*	*Estimate*	*P Value*	*Estimate*	*P Value*	*Estimate*	*P Value*	*Estimate*	*P Value*
Blockgroup Level Variance	0.01685	<0.0001	0.01505	<0.0001	0.01505	<0.0001	0.01421	<0.0001	0.01422	<0.0001
Residual ICC	0.00486	0.04	0.00456	0.05	0.00456	0.05	0.00430	0.06	0.00431	0.06

Among women with a histological grade of tumor reported, those with grades 3 or 4, compared to those with grades 1 or 2.

Age in years, centered at the median age of 59. Year of Diagnosis is centered at 1997.

Census blockgroup social class index standardized by subtracting median (272), dividing by the standard deviation (37.7).

Census blockgroup estimated tobacco spending is average per household, in units of $100, and is centered at median (7).

Significance of variance of the blockgroup-level random intercept calculated with the Wald *X* ^2^ test.

Significance of the residual intraclass (within blockgroup) correlation based on the likelihood ratio *X* ^2^ test.

Later stage at diagnosis (Table 3) is also less common among older cases, as well as women diagnosed more recently, and is more common among black cases. Women with aggressive tumor grade are also more likely to be diagnosed at a later stage. Area-level social class is protective for later stage diagnosis, but a statistically significant interaction term again indicates that this effect of area-level social class is less protective for African American women. Area-level tobacco spending is not statistically associated with later stage at diagnosis, for either white or black cases.

**Table 3:** **Multi-level random effects logistic regression model of individual and area-level factors associated with later stage diagnosis of breast cancer, among white and black women in Maryland, 1992–2003 (32,673 women in 3565 census blockgroups, Maryland cancer registry)**

	**Model 1**		**Model 2**		**Model 3**		**Model 4**		**Model 5**	
Fixed Effects	O.R.	95% CI	O.R.	95% CI	O.R.	95% CI	O.R.	95% CI	O.R.	95% CI
Intercept	0.32	(0.31,0.33)	0.32	(0.31,0.34)	0.32	(0.31,0.33)	0.33	(0.29,0.38)	0.33	(0.28,0.39)
Individual Level										
Age	0.99	(0.97,0.98)	0.99	(0.99,0.99)	0.99	(0.99,0.99)	0.99	(0.99,0.99)	0.99	(0.99,0.99)
Black Race	1.24	(1.17,1.32)	1.15	(1.08,1.22)	1.18	(1.11,1.26)	1.18	(1.10,1.26)	1.19	(1.10,1.28)
Aggressive Grade	2.20	(2.09,2.31)	2.19	(2.08,2.30)	2.19	(2.08,2.30)	2.19	(2.08,2.29)	2.19	(2.08,2.29)
Year of Diagnosis	0.98	(0.97,0.98)	0.98	(0.97,0.99)	0.98	(0.97, 0.99)	0.98	(0.97,0.99)	0.98	(0.97,0.99)
Census Blockgroup										
Social Class			0.89	(0.86,0.91)	0.87	(0.84,0.89)	0.96	(0.83,0.90)	0.86	(0.83,0.90)
Class*Black Race					1.09	(1.02, 1.16)	1.09	(1.03,1.16)	1.10	(1.03,1.17)
Tobacco Spending (per $100)							1.00	(0.98,1.02)	1.00	(0.97,1.02)
Tobacco*Black Race									1.00	(0.97,1.05)
Random Effects	*Estimate*	*P Value*	*Estimate*	*P Value*	*Estimate*	*P Value*	*Estimate*	*P Value*	*Estimate*	*P Value*
Blockgroup Level Variance	0.03343	<0.0001	0.02168	<0.0001	0.02091	<0.0001	0.02089	<0.0001	0.02083	<0.0001
Residual ICC	0.01066	0.001	0.0655	0.028	0.00632	0.032	0.00631	0.033	0.00629	0.033

Among women with a stage of diagnosis recorded, those whose SEER stage was 2–7, compared to stage 1.

Age in years, centered at the median age of 59. Year of Diagnosis is centered at 1997.

Census blockgroup social class index standardized by subtracting median (272), dividing by the standard deviation (37.7).

Census blockgroup estimated tobacco spending is average per household, in units of $100, and is centered at median (7).

Significance of variance of the blockgroup-level random intercept, calculated with the Wald *X* ^2^ test.

Significance of the residual intraclass (within blockgroup) correlation based on the likelihood ratio *X* ^2^ test.

Tumor size information was only analyzed for four years (1999–2003). There was no significant effect of year of diagnosis, and therefore this variable was not retained in the models (Table 4). African-American cases were significantly more likely to be diagnosed with a tumor greater than two centimeters in size, and older women were less likely to have larger tumors. Social class was protective for large tumors, with a statistically significant interaction term indicating this protective effect was less strong for black women. In addition to the area-level effect of social class, area-level tobacco spending was significantly associated with larger tumor size, with the odds of larger tumor increasing 3% with $100 of average annual household tobacco purchases above the average value of $700. This effect was not statistically different for white and black cases. In each model, we also examined a potential interaction between social class and tobacco spending, but these terms were not statistically significant and were not retained (data not shown).

**Table 4:** **Multi-level random effects logistic regression model individual and area-level factors associated with large tumor size at diagnosis for breast cancer among white and black women in Maryland, 1999–2003 (17,248 women in 3413 census blockgroups, Maryland cancer registry)**

	**Model 1**		**Model 2**		**Model 3**		**Model 4**		**Model 5**	
Fixed Effects	O.R.	95% CI	O.R.	95% CI	OR	95% CI	O.R.	95% CI	O.R.	95% CI
Intercept	0.61	(0.58,0.63)	0.63	(0.61,0.65)	0.64	(0.61,0.66)	0.50	(0.42,0.60)	0.48	(0.38,0.59)
Individual Level										
Age	0.99	(0.99,0.99)	0.99	(0.99,0.99)	0.99	(0.99,0.99)	0.99	(0.99,0.99)	0.99	(0.99,0.99)
Black Race	1.59	(1.47,1.71)	1.45	(1.34,1.56)	1.49	(1.37,1.61)	1.56	(1.34,l.70)	1.54	(1.41,1.69)
Census Blockgroup Level										
Social Class			0.87	(0.84,0.90)	0.84	(0.81,0.87)	0.87	(0.83,0.92)	0.88	(0.83,0.93)
Class*Black Race					1.14	(1.05,1.23)	1.10	(1.01,1.19)	1.09	(1.00,1.18)
Tobacco Spending (per $100)							1.03	(1.01,1.06)	1.04	(1.01,1.07)
Tobacco*Black Race									0.97	(0.92,1.03)
Random Effects	*Estimate*	*P Value*	*Estimate*	*P Value*	*Estimate*	*P Value*	*Estimate*	*P Value*	*Estimate*	*P Value*
Blockgroup Level Variance	0.04203	<0.0001	0.02145	<0.0001	0.01863	<0.0001	0.01487	<0.0001	0.01462	<0.0001
Residual ICC	0.01261	0.01	0.00648	0.12	0.00563	0.15	0.0045	0.21	0.00442	0.21

Among women whose tumor size at diagnosis was reported, those with tumors > 2 cm, compared to those with tumor size < = 2 cm.

Age in years, centered at the median age of 59. Year of Diagnosis is centered at 1997.

Census blockgroup social class index standardized by subtracting median (272), dividing by the standard deviation (37.7).

Census blockgroup estimated tobacco spending is average per household, in units of $100, and is centered at median (7).

Significance of variance of the blockgroup-level random intercept, calculated with the Wald *X* ^2^ test.

Significance of the residual intraclass (within blockgroup) correlation based on the likelihood ratio *X* ^2^ test.

The variance of the blockgroup-level random intercept term is significant in all models, supporting the use of a multi-level random intercept approach for these analyses [34,35]. In each set of analyses, the significance of the residual intraclass correlation is reduced as blockgroup level fixed effects are added [34,35]. On visual inspection, semivariograms of final regression model residuals did not exhibit any remaining spatial autocorrelation between blockgroups [36,37].

## Discussion

Our findings confirm associations seen in previous studies, but also raise some new questions and suggest areas for future study. Our results are similar to those seen in other populations, in terms of the relationship between both racial minority status and social disadvantage, and breast cancer characteristics related to worse outcomes [38,39]. In our review of global literature on this topic, 11 of the 12 studies found social class to be protective for adverse breast cancer characteristics.

However, our analyses suggest that social class resources are not equally protective for black and white women, in that black women received less protection from higher social class in regard to later stage at diagnosis, more aggressive histological grade, and larger tumor size. This is consistent with theories of social stratification which focus on intersectionality, or the combined effects of race, gender, and social resources. Intersectionality theory [40] would argue that the meaning of a certain social achievement, for example college graduation or white collar employment, cannot be determined out of context from other social factors, and will confer different social advantage for women than men, and minority compared to majority racial groups. In the case of these analyses, a myriad of behavioral, environmental, or early detection-related factors may differ more between socially advantaged and disadvantaged white communities than those in which black cases reside.

In addition, our analyses found an additional community-level characteristic, average household tobacco expenditures, was significantly related to larger tumor size and aggressive histological grade, even after adjusting for community-level social class. The effect was relatively modest, and increased risk for aggressive grade only 1% per $100 of annual household spending, and 3% per $100 for larger tumor size. However, the findings do suggest the possibility of an independent relationship of tobacco to biological indicators of breast cancer disparities.

Why would area-level tobacco spending predict individual residents’ breast cancers? One possible explanation suggests a direct causal pathway, in that this cross-sectional area-level indicator represents our best estimate of the case’s actual lifetime tobacco use, and that use directly influenced her cancer outcomes. A related explanation would suggest that these consumer expenditure data are indicators of communities with high levels of general tobacco use and exposure to second and third hand environmental tobacco toxins, which had an influence on breast cancer outcomes for residents. There is evidence from both cohort and case/control studies which support both active and passive smoking as risk factors for adverse breast cancer outcomes [24-27]. A third theory suggests that as tobacco use becomes more heavily concentrated among socially disadvantaged groups, that tobacco use is a proxy for other negative health characteristics of communities, ranging from unhealthful individual level behaviors to excess exposure to environmental risks, and these influence each resident’s cancer outcomes to varying degrees.

The relationships seen here between tobacco use and three different adverse breast cancer outcomes are suggestive, and merit further exploration. Only two of the three disease characteristics were significantly associated with tobacco spending. We might speculate that there was no statistically significant relationship between tobacco spending and stage of disease at diagnosis, because late stage diagnosis is strongly influenced by uptake of mammography screening, in addition to disease characteristics. Future studies should focus on identifying effects associated with active tobacco use versus exposure to tobacco, as well as distinguishing between pre- and post- menopausal breast cancers. Furthermore, issues of timing of exposures, and the periods of greatest influence across a woman’s life course are also important.

Our data used estimated spending on tobacco products as a proxy for tobacco use. Although pricing of tobacco products is not likely to vary significantly across a single State, purchasing patterns of products can only approximate how cigarettes are consumed in different tobacco user populations. For example, the informal resale of single cigarettes (“loosies”) in low income neighborhoods, resulting in a higher price per cigarette than when purchased in packs or cartons, may not be accurately reflected in these data. At the global level, as tobacco consumption changes, improving metrics and measurement of tobacco use and exposure at the population level is an important challenge for tobacco control research and policy.

Our analyses used data from a well-established registry in a racial and socio-economically diverse U.S. State. However, the absence of data on more detailed disease and tumor characteristics, including hormone receptor status, limits our ability to examine subgroup effects. The absence of staging, histological grading, or tumor size information in clinical records is a well-recognized limitation of population-based registries, and as our previous work has explored [41]. In the current analysis, black women were less likely than white women to have tumor size information, which may have attenuated the estimates of excess risk associated with race, but it is not likely to have significantly biased the analyses of grade or stage.

Additionally, we were limited by sample size to examining only two ethnic groups. Future work in diverse geographic regions, utilizing clinically detailed registries and tumor banks could build on these initial findings.

## Conclusions

Continued consideration of tobacco as both a social and biological agent in health necessitates approaches informed by sociological theories of intersectionality, which argue that social influences such as race, gender and social class must be considered holistically, rather than as individual factors. The findings from our research would suggest that social class has a specific meaning and impact by race.

Our results did not identify a significant interaction between tobacco spending and social class, nor variation in tobacco effect by race. However, more research within diverse populations and geographic settings is needed. Although historically rates of tobacco use have been lower among African Americans than whites in the United States, there is evidence suggesting that tobacco addiction may be more harmful for low resource populations, perhaps due to interactions with dietary or occupational exposure effects [42].

Comparisons both within and across social groups will continue to offer the most complete insight into the causes and potential solutions to breast cancer related disparities. Single geography studies are essential for locally relevant service and policy decisions, but also remain valuable for building the evidence base across geographies to understand the complex relationship between inherited risk and environmental exposures. Given the growing global burden of breast cancer, especially in low resource societies, primary prevention at both the behavioral and environmental level is emerging as a critical strategy. Geographically-informed analyses of cancer burden allow us to compare across cultures and societies, and identify both consistent social drivers of health disparities, as well unique factors which may be overlooked in larger investigations.

## Materials and methods

### Case data

With IRB approval, a data use agreement allowed the investigators to obtain records from the Maryland Cancer Registry on all breast cancers reported to the Registry between 1992 (the first year of analysis-quality data reporting) and 2003, the most recent year available at the time of the agreement (2008). Data on cases originating with the Veterans Administration, Vital Statistics death records, and reports back to Maryland from states without research release agreements were not released. Cases of male breast cancer, those with missing race /ethnicity, and those with race/ethnicity other than white or black were removed, as well as those missing key covariates (age, year of diagnosis, gender). Individual-level variables extracted from the Maryland Cancer Registry record were examined for completeness, and the following variables were retained for analysis: race, age at diagnosis, year of diagnosis, tumor histological grade at diagnosis, SEER stage at diagnosis [33], and (for years 1999–2003 only) tumor size in millimeters.

Geocoding was used to match each case by residential address to a latitude/longitude point location. Standard geocoding processes involved iterative address cleaning and reattempting geocoding. Addresses for cases which were not matched by software were manually reviewed, and for those cases determined to have a legitimate Maryland residential location, we used our previously developed imputation algorithm, to assign non-geocoded cases to a point location within their zip code, based on Census age-, race- and gender-specific population distribution patterns of all Census blocks within a zipcode. Each case in a zipcode whose specific location cannot be determined through geocoding is randomly assigned to a block within that zipcode, with probability of assignment based on population characteristics of that block, thus using what is known about the distribution of residents within the zipcode to improve the random assignment [32]. Imputation is most commonly needed for cases in rural areas whose mailbox or rural route address does not identify a geocodable point location. Imputation allows for full use of data across geographic areas, and avoids biasing useable data and therefore results towards urban cases.

### Area-level covariate data

Based on point location, each case record was linked to a Census block group location. Files of block group-level Census characteristics were obtained for the 1990 and 2000 Census. Based on our previous work [18], we selected the Census block group as a unit of population and geography best suited to examining small-area community-level social resource influences on cancer outcomes. Previous work (more details available in [18]) used factor analysis to identify a reliable four-item composite area-level measure of social class for Maryland, which created an index by summing together 1) percent high school graduates among persons age 26 and older, 2) percent of persons employed, among those actively seeking employment, 3) percent of the working population holding white collar jobs, and 4) per capita income, in $1000 units. To most accurately capture changing demographics in each block group across the time window, based on year of diagnosis, each case was assigned a value for each of the four measures which was a weighted combination of her block group’s Census values for 1990 and 2000. For example, a case reported in 1995 would be assigned a weighted combination of 0.5*1990 values + 0.5*2000 values. The commercially prepared Census data also adjusted for changes in block group boundaries between the 1990 and 2000 Census. Women diagnosed in 2001–2003 were assigned the values for 2000.

A measure of area-level tobacco product use was created based on data produced from the Consumer Expenditure Survey (CEX) of the Bureau of Labor Statistics (www.bls.gov/cex). The U.S. Census Bureau conducts the Consumer Expenditure Survey for the Bureau of Labor Statistics, gathering both continuous diary data of routine purchases and quarterly surveys on large purchases from a representative sample of US individuals and households, as well as sociodemographic descriptors of the consumer unit. Unlike the U.S. Census, these data, while geographically representative of the United States, are gathered from only 7000 consumer units. However, these data are combined with Census and other proprietary data by multiple geographic data vendors to produce area-level estimates for all US block groups. Unlike point-of-purchase or vendor-based sales data, which report what is purchased by all shoppers in a given store or geographic area, these estimates are of household and community-level spending behaviors, and are therefore less sensitive to the area-level retail environment or pricing.

We used the “Business Analyst” estimates produced by ESRI for use with ArcGIS software (www.ESRI.com), and used blockgroup-level estimates of the average spending on all tobacco products per household. We selected household rather than individual level spending estimates, because tobacco products are typically used predominantly by adults. CES data were not available with 1990–2000 Census boundary “crosswalks”, and therefore each case was assigned the 2000 value for the average household tobacco spending, in dollars per year, for her block group.

### Data analysis

Univariate and bivariate descriptive analyses examined the distribution of key case characteristics (age, diagnosis year, tumor grade, stage, and size) for the entire population and by race, as well as the distribution of the four social class variables and tobacco spending by block group and cases. Multivariate logistic regression models were used to estimate the effect of case-specific characteristics and area-level social class and tobacco spending on three different breast cancer characteristics associated with adverse outcomes: aggressive tumor histology, defined as histological grade 3 or 4, compared to grade 1 or 2, later stage diagnosis, defined as women diagnosed at SEER stage 2–7, compared to stage 1, and large tumor size at diagnosis, defined as women whose tumor size was reported as greater than two centimeters, compared to those with tumors less than or equal to two centimeters.

For each of the three outcomes, we estimated five different models, beginning with individual case characteristics (age, race, and year of diagnosis). Next a single area-level variable, the area-level social class index, was added. Next, in order to examine whether social class effects varied by race, the interaction of class and race was estimated. Then tobacco spending per block group was added to examine the additional effect, if any, of tobacco consumption. Finally, differential effects of tobacco spending by race were examined.

Multi-level logistic regression models were used to incorporate the geographically nested nature of the data, where more than one woman may reside within a block group, thus violating the independence assumptions of conventional models. A two-level random effects model was used with a random intercept term [35].

To reduce collinearity within multivariate models and aid in interpretation of interaction effects, the four-item index value for Census block group social class was standardized by subtracting the median, and dividing by the standard deviation, and the tobacco spending values were modeled in units of $100, and centered at the median. Values for case age at diagnosis and year of diagnosis were centered at their median values.

For each model, we report the odds ratios and 95% confidence intervals for the intercept term and individual and area-level fixed effects. Model diagnostics include estimates of the blockgroup level variance and intra-class correlation, as well as the related p values from the likelihood ratio-based statistical tests. This allows us to compare nested models for the significance of included random effects terms [34,35]. To test for any unexplained spatial autocorrelation among residuals, we examined spatial semivariograms of regression model residuals [36,37]. Geocoding and GIS linkages were conducted with ArcGIS (www.ESRI.com), imputation was conducted using the R programming language [43], and all multivariate regression modeling was conducted using the XTLOGIT program in STATA [35].
